# 1822. Assessing Trusted Sources and its Impact on Healthcare Accessibility for Safe Return to K-12 Schools in an Underserved Population

**DOI:** 10.1093/ofid/ofad500.1651

**Published:** 2023-11-27

**Authors:** Humza Agha, Jamee T Shelley, Sydney A Reyes, Summer M Reyes, Tyler Walsh, Tremayne Watterson, Ana V Torres, Brittany T Bonty, Christina Evans, Jasmine Prater, Samantha Hayes, Sara Malone, Albert Lai, Stephanie A Fritz, Jason Newland, Julie A Neidich, Ian T Lackey

**Affiliations:** Washington University in St. Louis School of Medicine, St. Louis, Missouri; Washington University School of Medicine, SAINT LOUIS, Missouri; Washington University in St. Louis School of Medicine, St. Louis, Missouri; Washington University School of Medicine, SAINT LOUIS, Missouri; Washington University School of Medicine in Saint Louis, St. Louis, Missouri; Washington University in St. Louis School of Medicine, St. Louis, Missouri; Washington University in St. Louis School of Medicine, St. Louis, Missouri; Washington University School of Medicine, SAINT LOUIS, Missouri; Washington University in St. Louis School of Medicine, St. Louis, Missouri; Washington University School of Medicine, SAINT LOUIS, Missouri; Washington University in St. Louis School of Medicine, St. Louis, Missouri; Washington University School of Medicine, SAINT LOUIS, Missouri; Washington University in St. Louis, St. Louis, Missouri; Washington University School of Medicine, SAINT LOUIS, Missouri; Washington University School of Medicine, SAINT LOUIS, Missouri; Washington University School of Medicine in St. Louis, St. Louis, Missouri; Washington University in St. Louis School of Medicine, St. Louis, Missouri

## Abstract

**Background:**

Participants of a COVID-19 school-based testing program in predominantly Black schools were asked to complete the NIH’s Common Data Elements (CDE) survey to help understand the demographic and social determinants of health of our testing population. The objective of this study was to assess if those completing the CDEs were reflective of our overall testing participants and to identify the trusted sources of these individuals

**Methods:**

All testing participants were offered the opportunity to complete a 150 question CDE survey after being enrolled in the testing portion of the study. Descriptive statistics described race/ethnicity, socioeconomic status, and vaccination status. Geospatial mapping utilizing addresses of participants was used to assess social vulnerability index among testing and CDE participants. Response options for trusted COVID19 source questions were given in a 5-point Likert scale, with answers ranging from “A great deal,” to “Not at all.”

**Results:**

A total of 477 CDE surveys were submitted among 2581 participants. When comparing the total participant population with the CDE respondent population, we see disparate numbers among Black and White participants (Fig. 1). Additionally, those completing CDEs were in lower SVIs (Fig. 2). Analysis of trusted sources for COVID19 information showed that 80% of White CDE respondents trusted their doctor/healthcare provider “a great deal,” as compared to 26% indicating “not at all” for Faith Leader. 51% of Black CDE respondents trust their doctor/healthcare provider “a great deal,” as compared to 25% indicating “not at all,” for the US Government. (Fig. 3)

Testing population vs. CDE population by race

**Figure:**
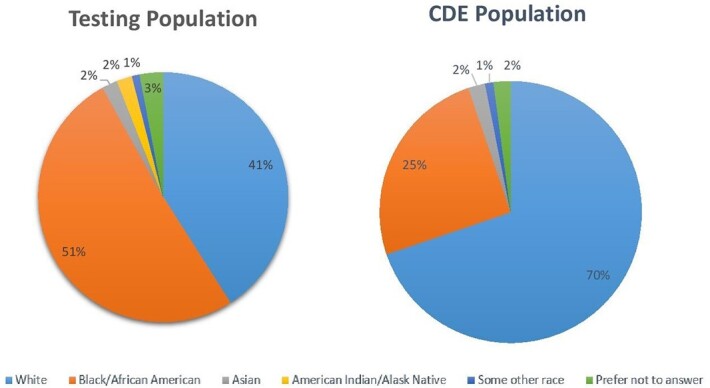

This graph shows our overall testing population and CDE population by race.

Mapping testing population vs. CDE population SVI

**Figure:**
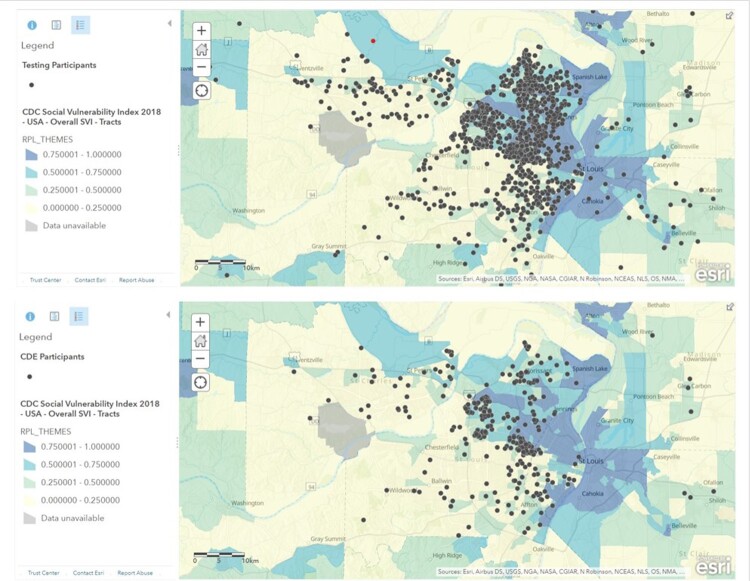

The addresses of our overall testing population and our CDE population were geospatially mapped against CDC's 2018 Social Vulnerability Index (SVI).

CDE trusted COVID19 sources by Race

**Figure:**
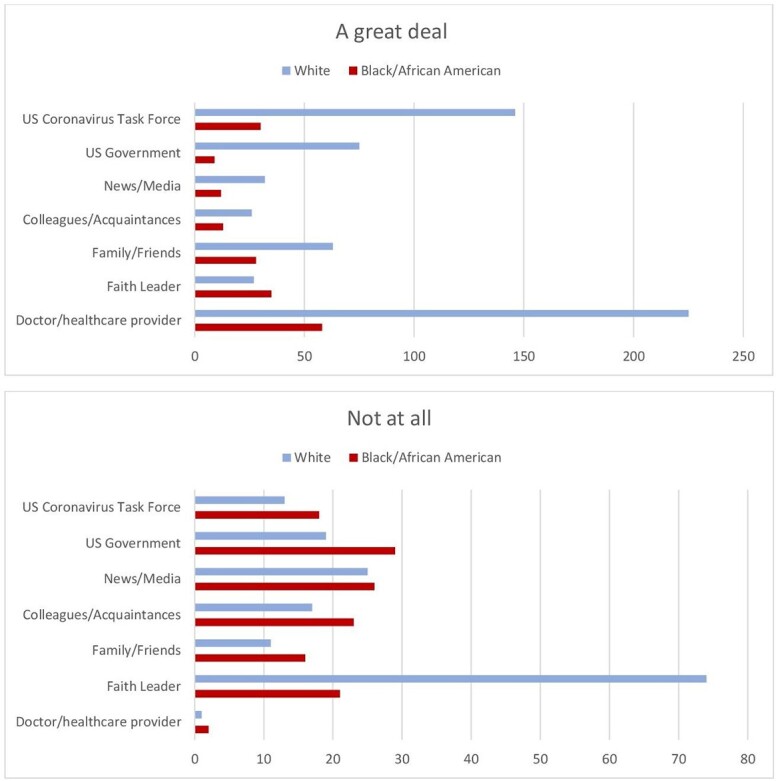

Responses from our testing population that completed CDEs based on trusted sources

**Conclusion:**

CDE respondents did not reflect our testing participants. Additionally, the most trusted sources among the population completing the CDEs were doctors/healthcare providers, and not as much faith leaders. While these data are important, they do not reflect the testing participants in our underserved community and need to be interpreted with caution. Use of CDEs are important but care needs to be taken when reporting the results.

**Disclosures:**

**Albert Lai, PhD**, Johnson & Johnson: Stocks/Bonds **Jason Newland, MD**, Moderna: Grant/Research Support|Pfizer: Grant/Research Support

